# Utilization of cuproptosis-related lncRNAs to predict the prognosis of pancreatic cancer patients and explore their roles in immune cell infiltration and prognosis evaluation

**DOI:** 10.1016/j.gendis.2024.101409

**Published:** 2024-09-06

**Authors:** Kaili Liao, Jinting Cheng, Xiajing Yu, Ziqian Liu, Xue Zhang, Yuxin Fu, Wenyige Zhang, Jie Liu, Feifei Teng, Yuxuan Xie, Xiwen Yan, Gaoquan Cao, Bing Sun, Hanqing Zhao, Jingyan Zhang, Xiaozhong Wang

**Affiliations:** a Jiangxi Province Key Laboratory of Immunology and Inflammation, Jiangxi Provincial Clinical Research Center for Laboratory Medicine, Department of Clinical Laboratory, The Second Affiliated Hospital, Jiangxi Medical College, Nanchang University, Nanchang, Jiangxi, China; b School of Public Health, Jiangxi Medical College, Nanchang University, Nanchang, Jiangxi 330006, China; c The 1^st^ Clinical Medical College, Jiangxi Medical College, Nanchang University, Nanchang, Jiangxi 330006, China; d Queen Mary College, Jiangxi Medical College, Nanchang University, Nanchang, Jiangxi 330006, China; e The 2^nd^ Clinical Medical College, Jiangxi Medical College, Nanchang University, Nanchang, Jiangxi 330006, China; f The 4^th^ Clinical Medical College, Jiangxi Medical College, Nanchang University, Nanchang, Jiangxi 330006, China

The prognosis of pancreatic cancer (PC) is difficult to predict^1^ and is extremely poor.^2^ Studies showed that cuproptosis was related to PC. The roles are not completely understood. It is considered that lncRNAs are closely associated with PC. We explored the relationship of curproptosis-related lncRNAs (CRLs) with the prognosis of PC patients and their potential role. We determined 19 prognostic CRLs through Pearson correlation analysis and univariate Cox regression analysis from 185 tumor samples. Subsequently, we constructed a predictive prognosis model for PC patients based on four CRLs and utilized the formula to calculate the risk score. The values for the area under the receiver operating characteristic (ROC) curve (AUC) were 0.699, 0.749, and 0.824 at 1 year, 3 years, and 5 years, respectively, which suggested that our risk model showed good predictive ability. Besides, we found the potential regulatory pathways by enrichment analysis, obtained the differences in immune microenvironment and drug sensitivity in different risk groups, and found that drug sensitivity in the high-risk group of other drugs was significantly lower. The prognostic model can accurately evaluate the prognosis for PC patients. These CRLs were related to the immune microenvironment, which can become underlying biomarkers for PC prediction, immunotherapy, and drug therapy.

We first obtained a dataset of PC from the TCGA database, which included 185 tumor samples. Based on previous studies, 19 cuproptosis regulators (ATP7A, ATP7B, DBT, DLST, FDX1, GCSH, GLS, LIAS, LIPT2, MTF1, NLRP3, PDHA1, PDHB, SLC31A) were selected for univariate Cox regression analysis, and 121 CRLs were identified (selection criteria: |Pearson R| > 0.4 and *P* < 0.001) (Fig. 1B and Table S1), and the correlation between them is shown by the Sankey diagram (Fig. 1A). The 19 CRLs were analyzed by LASSO-Cox regression (Fig. 1C, D), and MIR223HG, C1QTNF1-AS1, CASC8, PAN3-AS1 were selected to construct prognostic models by “Corplot” collaborative screening (Fig. S2B).

**Figure 1:**
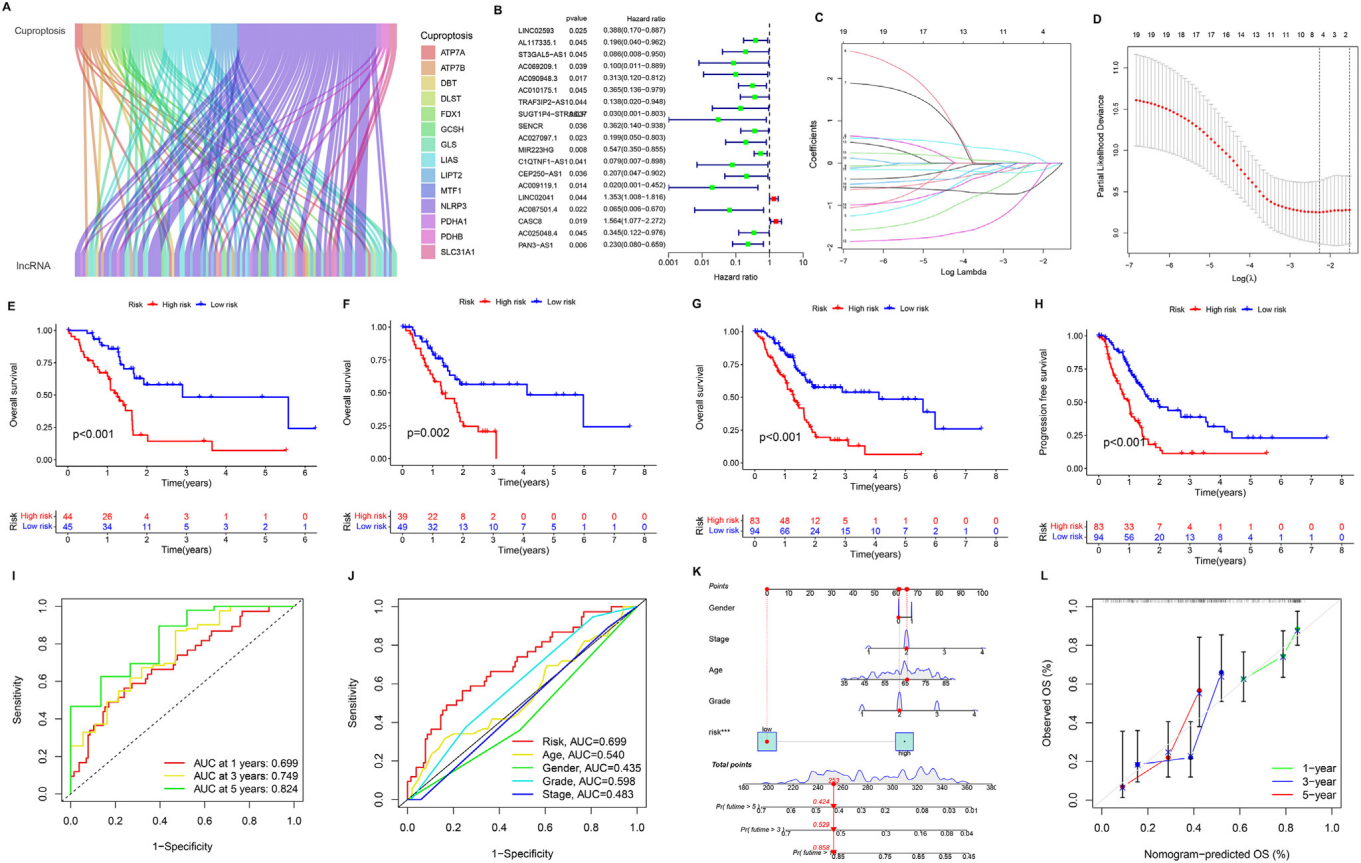
Construction and validation of a predictive prognosis model of pancreatic cancer based on cuproptosis-related lncRNAs. **(A)** The results of co-expression of cuproptosis-related genes and cuproptosis-related lncRNAs. **(B)** The results of Cox regression analysis with red representing high-risk lncRNAs and green representing low-risk lncRNAs. **(C)** LASSO regression screening of cuproptosis-related lncRNAs. **(D)** Trajectory of each independent variable. **(E)** Overall survival (OS) in the training group. **(F)** OS in the test group. **(G)** OS in all groups. **(H)** Progression-free survival (PFS) in all groups. **(I)** The 1-, 3-, and 5-year area under the receiver operating characteristic (ROC) curve (AUC) **(J)** The ROC curves representing risk scores and other clinical features. **(K)** The prognostic nomogram. **(L)** Calibration plot of predicted outcomes of 1-, 3-, and 5-year OS in patients.

We further analyzed the differences between the two groups in terms of risk score distribution, survival status, and survival time by dividing the patients into high-risk and low-risk groups according to the median risk score (Fig. S1A–I). The heat map showed that CASC8 exhibited significantly higher expression while MIR223HG, C1QTNF1-AS1, and PAN3-AS1 exhibited lower expression in the high-risk group. Previous studies also proved that CASC8 expressed higher in pancreatic adenocarcinoma tissues with more advanced grades and T-stages, and PAN3-AS1 expression dropped as detected by real-time PCR.^3^^,^^4^ In contrast, in the low-risk group, the opposite pattern of gene expression was observed.

To further validate our model, we performed a survival analysis for both risk groups. The Kaplan–Meier survival curve showed that patients in the high-risk group had significantly worse clinical outcomes (Fig. 1E, F) and shorter progression-free survival than those in the low-risk group (Fig. 1H). We also performed ROC analysis, which showed that the AUC values were 0.699, 0.749, and 0.824 at 1 year, 3 years, and 5 years, respectively (Fig. 1I), and our risk model showed better predictive power than other clinical characteristics (Fig. 1J). Moreover, the validation results from the ICGC dataset showed that the AUC values were 0.772, 0.683, and 0.650 at 1 year, 3 years, and 5 years, respectively (Fig. S2A). This indicates that the prediction performance of our model is good. In addition, the results of univariate and multivariate Cox regression analyses showed that the risk score had independent prognostic significance (Fig. S2C, D), and the risk score showed higher consistency compared with clinical characteristics such as age and gender (Fig. S2E). The prognostic model was further validated in all groups, training group, and test group. Principal component analysis showed that the expression level of CRLs could distinguish between the high- and low-risk groups (Fig. S3A, B), which indicated that the risk-scoring model constructed had high accuracy.

Next, we constructed a nomogram using gender, tumor tissue stage, age, tumor tissue grade, and risk score to predict the overall survival of PC patients (Fig. 1K). The calibration plot showed that the predicted overall survival was in good agreement with the observed 1-, 3-, and 5-year overall survival, indicating that the nomogram had good prediction performance (Fig. 1L). We then compared the survival status of patients with high and low risk in different stages of PC. The results showed that the survival time of the high-risk group was significantly lower than that of the low-risk group in stage I-II patients, but there was no significant difference in stage III-IV patients (Fig. S2F, G).

In addition, GO and KEGG enrichment analyses were performed to explore potential regulatory pathways (Fig. S3D, E), and a heat map was used to show the changes in immune function in different risk groups (Fig. S3F). The results showed that the expression level of immune function in the high-risk group was lower than that in the low-risk group. We also explored the genetic mutational profile of PC patients by selecting 15 potential mutational regulators in PC. In the high-risk group, regulator mutation was found in 176 out of 195 samples (90.26%) and TTN and TP53 mutations were the most frequent (Fig. S3G), and in the low-risk group, regulator mutations were observed in 148 out of 166 samples (89.16%) and TTN mutation was the most frequent (Fig. S3H).

Our study demonstrated variances in the sensitivity of 22 chemotherapeutic drugs at semi-inhibitory concentration IC50, as well as a correlation with risk scores (Fig. S4). By utilizing the pRophetic algorithm, we constructed a boxplot illustrating the disparity in IC50 sensitivity between low-risk and high-risk individuals for these drugs (Fig. S5). The results showed that epothilone B and thapsigargin were negatively correlated with the risk score, indicating that patients in the high-risk group were more sensitive to these drugs, and for other drugs, the sensitivity of the high-risk group was significantly higher than that of the low-risk group. Our findings provide valuable insights into immunotherapy and medication decisions for PC patients.

Our study also has certain limitations. Firstly, only 19 cuproptosis regulators were used to obtain the CRLs, which is not enough for the full understanding of the relationship between cuproptosis and PC. Secondly, external datasets are required to validate our conclusion. Thirdly, there are lack of relevant basic experiments to verify the targets and conclusions of our screening, which needs further research in the future. But in general, in this study, we identified cuproptosis-associated lncRNAs in PC. Based on four CRLs, our prognostic evaluation model could predict the prognosis of PC patients accurately. CRLs identified in our studies were related to the microenvironment. At the same time, these CRLs could be potential biomarkers for predicting overall survival in PC patients. This study can provide certain reference value for exploring the role of cuproptosis in PC, new insights for using cuproptosis-related lncRNAs to predict the prognosis of PC patients, and valuable views for immunotherapy and drug strategy of PC.

## Funding

This study was funded by the National Natural Science Foundation of China (No. 82160405).

## Data availability

All data is available. Please contact us to access if it is needed.

## CRediT authorship contribution statement

**Kaili Liao:** Formal analysis, Validation, Writing – original draft, Writing – review & editing. **Jinting Cheng:** Data curation, Writing – original draft. **Xiajing Yu:** Data curation, Formal analysis, Investigation, Validation. **Ziqian Liu:** Writing – original draft. **Xue Zhang:** Validation. **Yuxin Fu:** Formal analysis, Writing – original draft. **Wenyige Zhang:** Data curation. **Jie Liu:** Formal analysis. **Feifei Teng:** Formal analysis. **Yuxuan Xie:** Methodology. **Xiwen Yan:** Methodology. **Gaoquan Cao:** Methodology. **Bing Sun:** Visualization. **Hanqing Zhao:** Visualization. **Jingyan Zhang:** Visualization. **Xiaozhong Wang:** Funding acquisition, Project administration, Writing – review & editing.

## Conflict of interests

There is no conflict of interests in this study.

## Figures and Tables

**Figure S1:**
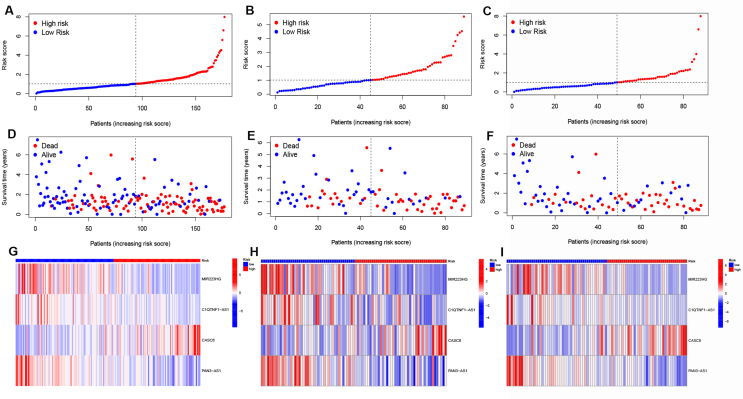
The survival status of patients and lncRNA characteristics across all groups. Risk curves and scatter plots represented the distributed survival status of patients with different risk scores; and the heatmap represented the characteristics of lncRNAs in the **(A)** training group, **(B)** test group, and **(C)** all groups.

**Figure S2:**
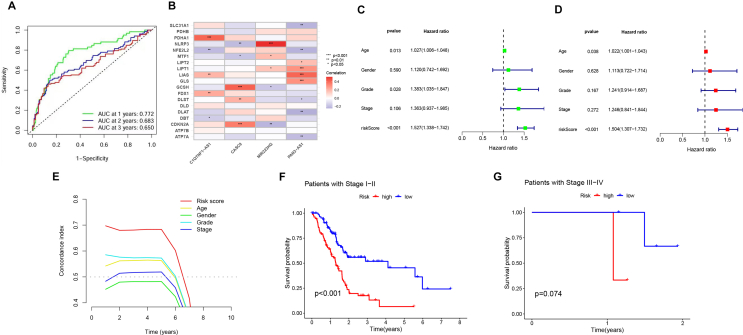
Model testing and patient prognosis analysis**. (A)** Validation results from the ICGC dataset: The 1-, 3-, and 5-year area under the receiver operating characteristic (ROC) curve (AUC). **(B)** The relationship between cuproptosis-related lncRNAs and cuproptosis-related genes for the signature. **(C, D)** The results of (C) univariate and (D) multivariate Cox regression analyses. **(E)** The C-Index curve represents the consistency index of the risk score. **(F, G)** The result of survival analysis on the low-risk group in (F) stage I-II and (G) stage III-IV patients.

**Figure S3:**
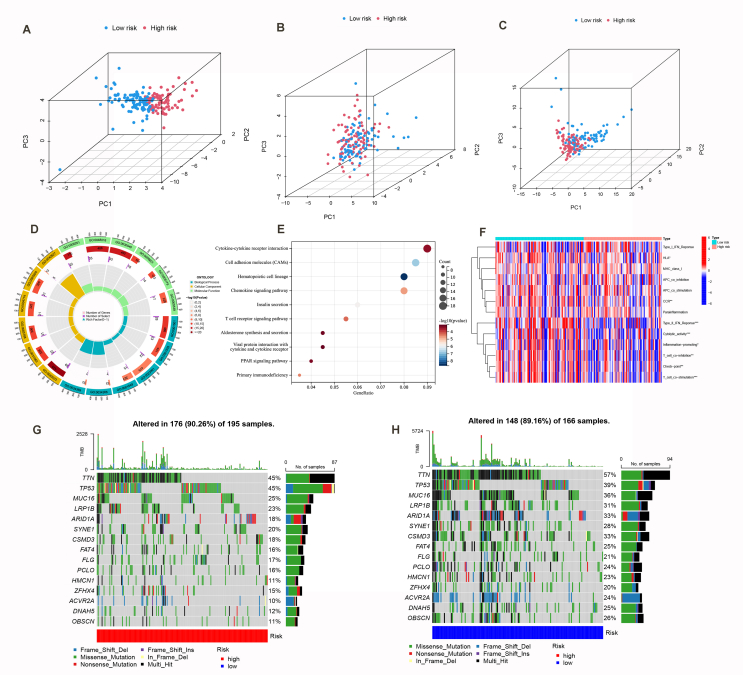
Immune function and gene mutations in the high- and low-risk groups. Principal component analysis (PCA) demonstrates the distribution of patients. **(A)** Risk lncRNAs. **(B)** Cuproptosis-related genes. **(C)** Cuproptosis-related lncRNAs. **(D)** The result of GO enrichment analysis. **(E)** The result of KEGG enrichment analysis. **(F)** Immune-related functions of the risk groups. **(G, H)** The waterfall plot revealed the top 15 mutation genes in the (G) high-risk group and (H) low-risk group.

**Figure S4:**
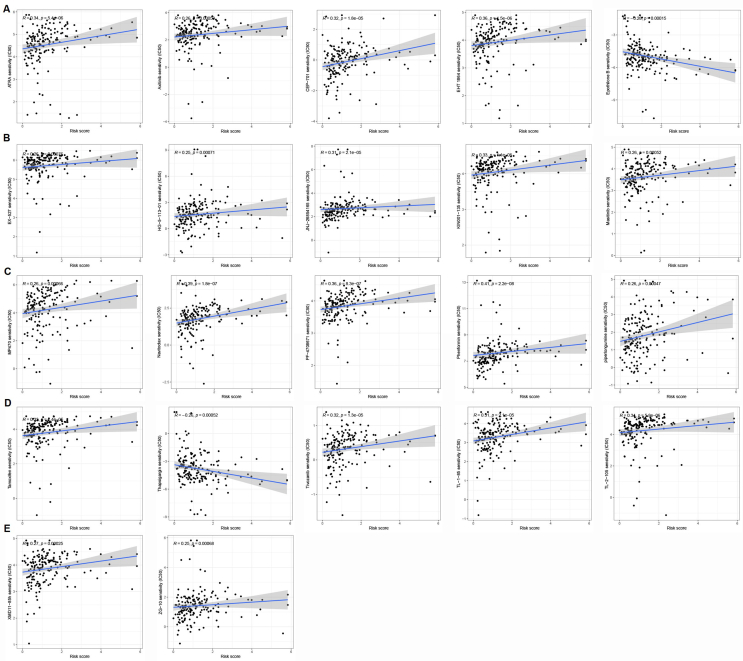
Correlation of chemotherapeutic drug sensitivity with risk scores.

**Figure S5:**
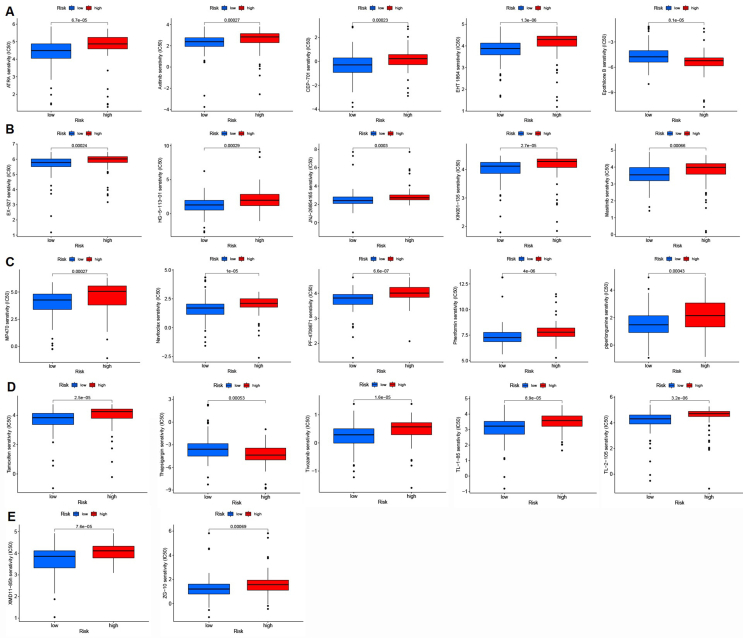
Results of drug sensitivity analysis.
