# Supplementary material for: Utilization of cuproptosis-related lncRNAs to predict the prognosis of pancreatic cancer patients and explore their roles in immune cell infiltration and prognosis evaluation
Source: Genes Dis. 2024 Sep 6;12(3):101409. doi: 10.1016/j.gendis.2024.101409 (PMC11795092; doi:10.1016/j.gendis.2024.101409)
Supplement: Multimedia component 1 [file mmc1.docx]

**Materials & Methods**

**Data collection**

We obtained the PAAD patient data sets, which is derived from the TCGA database (https://cancergenome.nih.gov/). In addition to those lacking expression substrates or corresponding clinical information, we also included cancer samples from 185 PAAD patients and extracted their lncRNA expression profiles. We obtained patient data from the ICGC database(https://dcc.icgc.org/) for external testing.According to existing studies, 19 copper death regulators have been identified, including ATP7A, ATP7B, DBT, DLST, FDX1, GCSH, GLS, LIAS, LIPT2, MTF1, NLRP3, PDHA1, PDHB and SLC31A.

# Bioinformatics analysis

Through the Pearson correlation coefficient analysis, we identified the lncRNA associated with copper death (|Pearson R| > 0.4, p < 0.001).We then used univariate Cox regression analysis (p < 0.05) to obtain survival curves for predicting clinical outcomes. We used "glmnet"R package to perform LASSO-Cox regression to further screen potential prognostic genes.The selection criteria of LASSO-COX regression were: p < 0.001 and the |coef| > 0. On the basis of meeting the criteria, the markers with large absolute value were combined together, and finally 4 markers were obtained. We used the cv.glmnet function for cross-validation screening, with the parameters set to: nfolds=10,alpha=1. All analyses were performed with the use of R version 4.1.0.

The Risk score was calculated based on the prescribed formula, where Risk score = ∑ Coef ∗EXP. In this formula, the term "Coef" denotes the coefficient, while "EXP" refers to the expression of each lncRNA associated with prognostic cuproptosis. Our complete calculation formula is: Risk score = (-0.407465804035788 * MIR223HG expression)-(1.83490450824287 * C1QTNF1-AS1 expression) + (0.485061146239699 * CASC8 expression) - (1.26424812361155 * PAN3-AS1 expression).PAAD patients were divided into two groups, namely a group with high risk and another group with low risk, according to their respective risk scores . Kaplan-Meier survival curves were generated using the "survminer" software in the R programming language., and ROC curves were generated utilizing the "timeROC" software

Use the "limma" package to identify genes with differential expression across various risk categories( | log2FoldChange | > 1.5, p<0.01). Subsequently, the software package "clusterprofiler" was utilized to conduct enrichment analysis using GO and KEGG for the genes that exhibit differential expression. We constructed column charts using the "rms" software package.The distribution of patients with high and low risk scores was assessed by performing Principal Component Analysis (PCA) using the scatterplot3d package.

The VarScan2 annotation file downloaded from the TCGA database was utilized to calculate the tumor mutation burden (TMB) of the sample through the VarScan2 channel somatic mutation call workflow, and genes that have undergone mutations were visually examined utilizing the maftools software suite. The pRRophetic R package was employed for evaluation the IC50 values of 22 chemotherapeutic drugs at a semi-inhibitory concentration, and the differential analysis of drug sensitivity was visualized in the form of graphical representations known as box plots.

# Quantitative analysis

Pearson correlation coefficient had been utilized for evaluating the association between regulators involved in cuproptosis and long non-coding RNAs (lncRNAs). When the absolute correlation coefficient exceeded 0.4, while the p-value was found to be below the significance level of 0.001,the lncRNA could be considered to be significantly associated with copper mortality. By utilizing Kaplan-Meier (KM) analysis, we evaluated the overall survival outcomes rates among subgroups.The accuracy of lncRNA features in both the training and testing sets was assessed using receiver operating characteristic (ROC) curve analysis. The risk scores among subgroups were compared using a t-test, while the chi-square test was employed for inter-group categorical variable comparisons. The Pearson correlation test was employed to examine the correlations among different subtypes. Cox regression analyses, both univariate and multivariate, were employed to explore the independent predictive risk score's numerical worth and other clinical characteristics on prognosis. A nomogram prediction model was constructed derived from multiple predictive factors like gender, stage of tumor tissue , age, and tumor tissue grade, and the total score was calculated to visually anticipate the 1-, 2-, and 3-year survival rates of pancreatic cancer patients. The methodology for calibration was used to verify the predictive function of the nomogram model on OS outcomes. We conducted statistical analysis using R software. The statistical significance of a p-value below 0.05 was established in our study.
