# Supplementary material for: Utilization of cuproptosis-related lncRNAs to predict the prognosis of pancreatic cancer patients and explore their roles in immune cell infiltration and prognosis evaluation
Source: Genes Dis. 2024 Sep 6;12(3):101409. doi: 10.1016/j.gendis.2024.101409 (PMC11795092; doi:10.1016/j.gendis.2024.101409)
Supplement: Multimedia component 2 [file mmc2.docx]

**Tables**

**Table S1** Results of the univariate cox regression analysis.

| ID | HR | HR.95L | HR.95H | *P* value |
| --- | --- | --- | --- | --- |
| LINC02593 | 0.388369219786867 | 0.170084319534414 | 0.886799272800347 | 0.0247588042693771 |
| AL117335.1 | 0.196096794520322 | 0.0399921576456138 | 0.961537338442723 | 0.0446098966621057 |
| ST3GAL5-AS1 | 0.0856688588651934 | 0.00772414999352438 | 0.950156766170683 | 0.0453265331788612 |
| AC069209.1 | 0.0997772376916662 | 0.0112012766284139 | 0.888782367549563 | 0.0388632961512907 |
| AC090948.3 | 0.312592115039537 | 0.120308939742505 | 0.812190936052019 | 0.0169883355275256 |
| AC010175.1 | 0.364595309178885 | 0.135777106099882 | 0.979029110971474 | 0.0452828559074976 |
| TRAF3IP2-AS1 | 0.138330903576578 | 0.0201867795613008 | 0.947919346233729 | 0.0439641929919562 |
| SUGT1P4-STRA6LP | 0.030309208246944 | 0.00114411207827372 | 0.802935413410465 | 0.0365063741288716 |
| SENCR | 0.361818337876962 | 0.139596367182869 | 0.937793097814321 | 0.0364262020050707 |
| AC027097.1 | 0.19946945047089 | 0.0495735016970665 | 0.802607447710585 | 0.0232362747561745 |
| MIR223HG | 0.547193106639397 | 0.350176918251314 | 0.855054346382669 | 0.00810783833076278 |
| C1QTNF1-AS1 | 0.0792635036376181 | 0.00699526436232258 | 0.898136608353241 | 0.0406871658678996 |
| CEP250-AS1 | 0.206679520306238 | 0.0473559860483821 | 0.902027973198035 | 0.0359832378659385 |
| AC009119.1 | 0.0204630440465748 | 0.000925434180895616 | 0.452475368098914 | 0.0138172837278757 |
| LINC02041 | 1.3526658853166 | 1.00762903228258 | 1.81585180525665 | 0.0443732084734693 |
| AC087501.4 | 0.0654491233223043 | 0.00639288535751152 | 0.670055460735747 | 0.0215995463451599 |
| CASC8 | 1.56447740241569 | 1.07713654541012 | 2.27231129897039 | 0.0187655519372318 |
| AC025048.4 | 0.344959673050133 | 0.121873181001993 | 0.976401658285333 | 0.044968307182629 |
| PAN3-AS1 | 0.229993358109988 | 0.0802575251191758 | 0.659090156295771 | 0.00621765448767954 |
